# The Role of Biphasic Shocks for Transthoracic Cardioversion of Atrial Fibrillation

**Published:** 2005-10-01

**Authors:** Simon J Walsh, Ben M Glover, Jennifer AA Adgey

**Affiliations:** Regional Medical Cardiology Centre, Royal Victoria Hospital, Belfast

**Keywords:** Atrial fibrillation, defibrillation, direct current cardioversion, biphasic waveform

## Abstract

The modern generation of transthoracic defibrillators now employ impedance compensated biphasic waveforms. These new devices are superior to those with monophasic waveforms and practice is currently switching to biphasic defibrillators for the treatment of both ventricular and atrial fibrillation. However, there is no universal guideline for the use of biphasic defibrillators in direct current cardioversion of atrial fibrillation. This article reviews the use of biphasic defibrillation waveforms for transthoracic cardioversion of atrial fibrillation.

## Introduction

Electrical cardioversion is a commonly performed procedure for the treatment of atrial fibrillation (AF) that has been successfully employed since the 1960’s [1]. Early devices used both alternating and direct current for defibrillation. However, by the 1960’s it was apparent that alternating current was more detrimental to the heart and direct current has been used in defibrillators since. Direct current cardioversion (DCC) was performed with critically damped monophasic waveforms for over 2 decades. However, these are produced with inductors used in conjunction with capacitors [2]. Inductors are electrical components that are too large for implantable devices. As interest in implantable defibrillators increased, research was subsequently driven towards truncated capacitor-based discharges. These waveforms were simple to produce, and improved capacitor technology facilitated their production in suitably sized implantable devices. We have described the production of these waveforms previously [3].

It was recognised subsequently that biphasic waveforms offered distinct advantages over monophasic waveforms in ventricular defibrillation (in terms of superior or equivalent efficacy at lower peak voltage, current and total delivered energy). These results stimulated further research into biphasic waveforms for defibrillation of AF, where similar results were reported for internal and external cardioversion.

## Why choose biphasic waveforms for transthoracic DCC of AF?

Some physicians have questioned whether biphasic waveforms offer significant advantages over monophasic defibrillators. It has been noted that monophasic waveforms result in similar rates of cardioversion by the maximum energy of a treatment protocol. It is already known that DCC does not result in cardiac damage at currently applied energies [4]. In addition, the use of biphasic waveforms does not appear to reduce the early recurrence of atrial fibrillation [5].

Many studies have shown that lower energy impedance compensated biphasic (ICB) waveforms are equivalent or superior to the higher energy monophasic waveforms (Table 1) [5-13]. These new devices are successful with fewer shocks [7], will offer successful cardioversion to patients where monophasic shocks have failed [14] and may obviate the need for transvenous cardioversion [15]. Skin burns are less common with biphasic waveforms[7] with the likelihood of a skin burn increasing proportionally with the total energy delivered with both monophasic [7] and biphasic devices [16]. In addition, biphasic waveforms have been shown to cause less muscle damage [9] and do not cause elevation of myoglobin and cardiac troponin I levels after DCC [17]. In summary, success can be achieved with biphasic waveforms at lower energy and with minimal deleterious effects for the patient.

Manufacturers are no longer producing monophasic defibrillators. These older devices will be superseded and replaced by those with biphasic waveforms. This will result in an inevitable shift in practice toward biphasic DCC of atrial arrhythmias.

## Electrode positioning in DC Cardioversion

Antero-posterior (AP) and antero-apical (AA) electrode positions are commonly employed during DCC of AF. There has been considerable debate as to whether any one position is optimal. Historically, AP configurations have been considered to provide a better “shock vector” through the atria compared with the AA configuration [18],[19]. Several antero-posterior configurations were described and theories were proposed to explain the advantages of these electrode positions, with a right anterior to left posterior configuration suggested where the underlying pathology involves both atria, whilst a left anterior to posterior configuration is better when the left atrium was primarily affected [19]. However, it is known that only a small percentage (~4%) of current delivered by the transthoracic route reaches the heart in an AA configuration [20], whilst the majority of current is shunted around the heart (around the thoracic cage or through the lungs). The path taken by current using AP electrodes is not described, and small variations in electrode positions may have a very small influence on outcome. Kerber had previously indicated that there was no significant difference between apex-anterior, apex-posterior and anterior-posterior configurations with monophasic waveforms achieving greater than 90% success in all groups [21]. Two studies have suggested that the use of an AP configuration may be superior when monophasic waveforms are employed for DCC of AF [22,23]. However, neither measured transthoracic impedance (TTI) with the varying electrode configurations.

TTI is known to be an important determinant of both atrial [24] and ventricular defibrillation [25]. Modern biphasic defibrillators compensate for differing transthoracic impedances. These devices alter the delivered waveforms in order to deliver similar defibrillation energy “doses” to patients at extremes of transthoracic impedance. Thus those with a low TTI (typically smaller and lighter) receive a limited voltage and current to deliver a set energy, whilst those with a high TTI (larger and heavier patients) receive more voltage and current to deliver the same given energy to the heart.

Kirchhof and colleagues have recently examined the midline AP configuration with non-impedance compensated monophasic and impedance compensated biphasic waveforms using hand-held paddles (a factor that is known to reduce TTI) [26]. This position was reported to be highly efficacious [26]. TTI was not reported. To date, AA and various AP configurations have been described with biphasic waveforms and the results are highly efficacious when a modern ICB defibrillator is employed. We have recently compared AA and AP electrode positions using a biphasic waveform and found that the AP position was associated with a lower TTI in a large number of patients. However, when a modern biphasic defibrillator with impedance compensation was employed for DCC, there was a trend towards an improved outcome with the AA position [27]. Our results suggest that reducing the influence of TTI with a modern device, and the utilisation of a biphasic waveform reduces the effect and importance of electrode position. We currently use the AA position for DCC in our institution.

## Energy selection

There are 2 distinct approaches to energy selection for DCC. Some physicians feel that it is best to select the highest possible energy in order to maximize the chance of initial success, minimize the number of shocks and lessen the exposure to sedative agents. Others prefer to follow an escalating energy protocol. Lown’s rationale for this approach was to minimise post-shock arrhythmia [18]. This method also allows cardioversion at the lowest energy for each individual patient and may prevent high cumulative doses in some.

At present each manufacturer recommends different energy selection protocols for their devices. Whilst lower energy shocks may be efficacious in patients with AF of short duration (1 week or less), published studies show that shocks of ≥150 J will result in a success rate of ~80% (Table 2). Two manufacturers offer the capability of higher energy biphasic waveforms (up to 360 J). Current evidence suggests that the majority of patients will be successfully cardioverted by 200 J shocks, and it appears that only a very small percentage of patients benefit from higher energy biphasic shocks. In addition, preliminary results from an ongoing multicentre study demonstrated that first shock success was significantly higher and fewer shocks were administered when a non-escalating energy selection protocol starting at 200J was employed (compared with an escalating protocol starting at 100J, maximum energy 200J) [28].

## What is the place of direct current cardioversion in the era of the rate control strategy?

Recent clinical trials have demonstrated that a rate control and anticoagulate strategy is acceptable for many patients with AF. The AFFIRM [29] and RACE [30] studies demonstrated that aggressive pursuit of sinus rhythm is not necessary in many patients. Nevertheless, many patients with AF will be acutely or chronically symptomatic necessitating cardioversion. Furthermore, those with a precipitating cause may have their arrhythmia abolished for the long term when the acute insult is resolved. Therefore, DCC will remain an important therapeutic option for a large proportion of patients with AF.

## Conclusions

Direct current cardioversion of AF using biphasic waveforms is highly efficacious and is superior to monophasic waveforms. All manufacturers’ devices have been shown to be effective for this procedure. The choice of electrode position appears to be less important when impedance compensated biphasic waveforms are employed for DCC. An initial energy of at least 150 J should be selected for DCC of AF, although only a small minority of patients will benefit from shocks of greater than 200 J.

## Figures and Tables

**Table 1:**
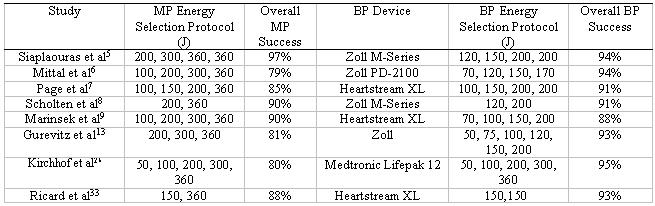
Studies comparing monophasic and biphasic devices for DCC in atrial fibrillation

MP: Monophasic BP: Biphasic

**Table 2:**
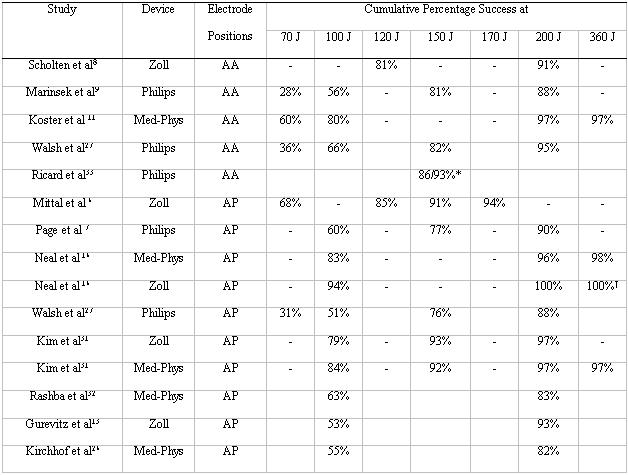
Evidence for energy levels and pad positions in biphasic direct current cardioversion of atrial fibrillation

*2 shocks delivered at same energy †Cross-over from Med-Phys to Zoll AA: Antero-apical Med-Phys: Medtronic-Physiocontrol AP: Antero-posterior
